# Supplementary material for: Single-cell RNA-sequencing reveals early mitochondrial dysfunction unique to motor neurons shared across FUS- and TARDBP-ALS
Source: Nat Commun. 2025 May 19;16:4633. doi: 10.1038/s41467-025-59679-1 (PMC12089458; doi:10.1038/s41467-025-59679-1)
Supplement: Supplementary file 2 — Description of Additional Supplementary Files [file 41467_2025_59679_MOESM2_ESM.pdf]

Description of Additional Supplementary Files

Supplementary Data 1. Literature review of clinical features in ALS-FUS.

Supplementary Data 2. Off-target analysis of top 5 homology regions for the iPSC lines in the DF6-9-9T.B background.

Supplementary Data 3. Differentially expressed genes (DEG) between cell types (motor neurons, V2 interneurons and other interneurons) for each individual cell line (control, FUS KO, FUS R495X, FUS P525L heterozygous and homozygous, TDP-43 M337V) from DESeq2.

Supplementary Data 4. Differentially expressed genes (DEG) between control and mutant cell lines (FUS KO, FUS R495X, FUS P525L heterozygous and homozygous, TDP-43 M337V) for each cell type (motor neurons, V2a interneurons and other interneurons) from DESeq2.

Supplementary Data 5. Differentially expressed gene sets of biological processes between control and mutant cell lines (FUS KO, FUS R495X, FUS P525L heterozygous and homozygous, TDP-43 M337V) for each cell type (motor neurons, V2a interneurons and other interneurons) from GSEA.
