# Supplementary material for: Single-cell RNA-sequencing reveals early mitochondrial dysfunction unique to motor neurons shared across FUS- and TARDBP-ALS
Source: Nat Commun. 2025 May 19;16:4633. doi: 10.1038/s41467-025-59679-1 (PMC12089458; doi:10.1038/s41467-025-59679-1)
Supplement: Supplementary file 8 — Reporting Summary [file 41467_2025_59679_MOESM8_ESM.pdf]

nature portfolio

Corresponding author(s): Hedlund and Ruepp

Last updated by author(s): Mar 30, 2025

Reporting Summary

Nature Portfolio wishes to improve the reproducibility of the work that we publish. This form provides structure for consistency and transparency in reporting. For further information on Nature Portfolio policies, see our Editorial Policies and the Editorial Policy Checklist.

Statistics

For all statistical analyses, confirm that the following items are present in the figure legend, table legend, main text, or Methods section.

n/a	Confirmed	
[ ]	[x]	The exact sample size (n) for each experimental group/condition, given as a discrete number and unit of measurement
[x]	[ ]	A statement on whether measurements were taken from distinct samples or whether the same sample was measured repeatedly
[ ]	[x]	The statistical test(s) used AND whether they are one- or two-sided<br>*Only common tests should be described solely by name; describe more complex techniques in the Methods section.*
[x]	[ ]	A description of all covariates tested
[ ]	[x]	A description of any assumptions or corrections, such as tests of normality and adjustment for multiple comparisons
[ ]	[x]	A full description of the statistical parameters including central tendency (e.g. means) or other basic estimates (e.g. regression coefficient) AND variation (e.g. standard deviation) or associated estimates of uncertainty (e.g. confidence intervals)
[ ]	[x]	For null hypothesis testing, the test statistic (e.g. F, t, r) with confidence intervals, effect sizes, degrees of freedom and P value noted<br>*Give P values as exact values whenever suitable.*
[x]	[ ]	For Bayesian analysis, information on the choice of priors and Markov chain Monte Carlo settings
[x]	[ ]	For hierarchical and complex designs, identification of the appropriate level for tests and full reporting of outcomes
[x]	[ ]	Estimates of effect sizes (e.g. Cohen's d, Pearson's r), indicating how they were calculated

Our web collection on statistics for biologists contains articles on many of the points above.

Software and code

Policy information about availability of computer code

Data collection	All software for data collection is publicly or commercially available and detailed in the methods section. Software to operate machines (Seahorse XF96 anlyzer: LSM800-Airy, LSM700, AxioObserver 7, Harmony 7) were recorded in the raw data files or analysis files provided. The raw and processed sequencing data is placed the Gene Expression Omnibus (GEO) with accession GSE226482. For the high content imaging on the Opera Phenix, the experiment and analysis files were provided on figshare. The other raw experimental data is on figshare as described in the data availability section.

Data analysis	All software for data anlysis is publicly or commercially available and described in the methods and code availability section. Meta-analysis of published clinical features of FUS-ALS cases was performed in R 4.2.0 with packages tidyverse 2.0.0, readxl 1.4.2, survminer0.4.9, survival 3.5-3, ggrepel 0.9.3, ggpubr 0.6.0, ggprism 1.0.4 (https://github.com/csdaw/ggprism). The analysis workflow and our custom code is here: https://github.com/schwi24/Schweingruber_et_al_2023/ALS_FUS_meta_analysis. Processing and mapping of sequencing reads was performed with STAR (2.7.0e) and rpkmsforgenes.py (PMID: 20011106) with gene annotations from the human genome from Ensembl 95 (GRCh38.95). Single-cell RNA-Seq analysis was performed in R 3.6.1 with packages Seurat 4.2.0, DESeq2 1.24.0 , fgsea 1.8.0, pheatmap 1.0.12, Vennerable 3.1.0, venn 1.11, ggplot2 3.4.1, ggupset 0.3.0. Gene ontologies were retrieved from MsigDB collection (C5, GO-BP). We published our scRNA-Seq analysis work flow and custom code here: https://github.com/NijssenJ/Schweingruber2023. Ingenuity pathway analysis (Qiagen) used the knowledge reference set Q4 2022. Structures were rendered with Chimera 1.16, build 42360. Image analysis was performed in Fiji 2.9.0 (PMID: 22743772) with the plugin Bio-Formats. Mitochondrial traces were detected in Fiji 2.9.0 with plugins Bio-Formats, TrackMate 7.0.0 (PMID: 35654950), TensorFlow 1.14.0 CPU, StarDist 0.8.3 (doi:10.1007/978-3-030-00934-2_30), and using the script mitotracker.py. Mitochondrial traces were analyzed in R 4.2.0 with packages tidyverse 2.0.0, tidygraph 1.2.3, ggraph 2.1.0, igraph 1.4.2, mitotrackR (https://github.com/schwi24/mitotrackR), ggcyclical (https://github.com/schwi24/ggcyclical), ggprism (https://github.com/csdaw/ggprism). The analysis workflow and our custom code is here: https://github.com/schwi24/Schweingruber_et_al_2023/Mitotracker. All R packages are available on CRAN unless indicated otherwise. We used renv 0.17.2 or later for version control of R packages.

For manuscripts utilizing custom algorithms or software that are central to the research but not yet described in published literature, software must be made available to editors and reviewers. We strongly encourage code deposition in a community repository (e.g. GitHub). See the Nature Portfolio guidelines for submitting code & software for further information.

Data

Policy information about availability of data

All manuscripts must include a data availability statement. This statement should provide the following information, where applicable:

- Accession codes, unique identifiers, or web links for publicly available datasets
- A description of any restrictions on data availability
- For clinical datasets or third party data, please ensure that the statement adheres to our policy

We have deposited all raw and processed sequencing data generated in this study on the NCBI Gene Expression Omnibus (GEO) under the accession number GSE226482. The C9orf72-ALS bulk RNA sequencing data was retrieved directly from the authors of the study (PMID: 33398403). Scans of fluorescent western blots, raw imaging files from confocal microscopy, the analysis files from Opera Phenix, qPCR data sets, and Seahorse assay result files with the following access links:

- Fig. 2a, https://figshare.com/s/9b92ae039360887d64ca;
- Fig. 2b-c, https://figshare.com/s/88c81e2e74e61a65b44d;
- Fig. 3d, https://figshare.com/s/2d7796e8ac90ee03075d;
- Fig. 3e-f, https://figshare.com/s/ee0947a5b9471a4b03f0;
- Fig. 5h-l, https://figshare.com/s/3ff566885092b09ec84f;
- Fig. 5m-n, https://figshare.com/s/97e16d3c225363385ecd;
- Fig. 6b-e, https://figshare.com/s/c923c637de679d6d87bb;
- Fig. 6f-h, https://figshare.com/s/c5b01718ea65968a967c;
- Supplementary Fig. 2c, https://figshare.com/s/8b4cda868991bf273bec;
- Supplementary Fig. 2d, https://figshare.com/s/39336063210c061fbed1;
- Supplementary Fig. 3b, https://figshare.com/s/821271881eebc7e1439e;
- Supplementary Fig. 3d, https://figshare.com/s/c25d0e50172144a23e7a;
- Supplementary Fig. 3j, https://figshare.com/s/0aa70000fec22559ee6c;
- Supplementary Fig. 4c, https://figshare.com/s/bbcf11d1f03cc50191b0;
- Supplementary Fig. 5a, https://figshare.com/s/4a0924eb6a07ff86394a;
- Supplementary Fig. 5b, https://figshare.com/s/a29d5bcb363c22758971;
- Supplementary Fig. 5c & 6b, https://figshare.com/s/33fdacf25a707e26bfcd;
- Supplementary Fig. 9d-g, https://figshare.com/s/25f3a49fa8f957d6441d;
- Supplementary Fig. 9h, https://figshare.com/s/b63363ea4fbe7e36fa5f.

Human research participants

Policy information about studies involving human research participants and Sex and Gender in Research.

Reporting on sex and gender	n.a.
Population characteristics	n.a.
Recruitment	n.a.
Ethics oversight	n.a.

Note that full information on the approval of the study protocol must also be provided in the manuscript.

Field-specific reporting

Please select the one below that is the best fit for your research. If you are not sure, read the appropriate sections before making your selection.

☒ Life sciences ☐ Behavioural & social sciences ☐ Ecological, evolutionary & environmental sciences

For a reference copy of the document with all sections, see nature.com/documents/nr-reporting-summary-flat.pdf

Life sciences study design

All studies must disclose on these points even when the disclosure is negative.

Sample size	No sample size estimations were performed. Independent neuronal differentiation rounds were considered independent biological replicates (n) and wells considered technical replicates. For single-cell RNA-Seq, neuronal cultures from a total of four independent differentiation rounds were used. For quantitative metabolic assays (Seahorse metabolic flux), motor neurons from several independent differentiation rounds ($n \geq 3$) were used with several wells per line each. For live-cell imaging to assess mitochondrial motility, motor neurons from several individual differentiation rounds were recorded in at least three technical replicates per line, namely control n = 4, FUS KO n = 5, FUS R244C n = 3, FUS R495X n = 2, FUS P525L heterozygous n = 4, FUS P525L homozygous n = 5, TARDBP M337V n = 5. For other quantiative microscopy, motor neurons from n = 3 individual differentiation rounds were used in at least three technical replicates wells.
Data exclusions	For single cell RNA-Seq, individual cells that did not pass quality control thresholds were excluded from the differential gene expression analysis (< 100,000 mapped reads; < 5000 detected genes at a cutoff of < 1 RPKM).<br>For quantitative metabolic assays (Seahorse metabolic flux) of motor neurons, wells with detached cultures were excluded from the analysis.<br>For mitochondrial motility analysis in motor neurons, individual movement steps that were orthogonal to the main axonal axis were excluded from the analysis.
Replication	We provide all experimental data and code to replicate the analysis. We have not reproduced our findings in other cell types or model systems but provide all experimental parameters for reproduction in the materials and methods section.We discuss similarities and discrepancies with published results extensively for cross-validation.
Randomization	Randomization is not applicable to this study. Experimental groups consist of isogenic cell lines with unique mutations. For quantitative metabolic assay, positions of cell lines in the plate layout were cyclically permutated to account for evaporation and oxygenation differences in edge wells. For microscopy, bias in manual selection of recording regions was accounted by choosing 3-4 regions as technical replicates.
Blinding	Blinding was not performed. Cell culture of cell lines in experimental groups was performed in parallel. Automation was used to remove user bias if possible and quantitative measures were recorded by machines. Analysis of RNA-Seq data was scripted. The same microscopy settings were used across experimental datasets. Image analysis was scripted in Fiji, python, and R with the same parameters for all experimental recordings that were established with separate set-up recordings/datasets.

Reporting for specific materials, systems and methods

We require information from authors about some types of materials, experimental systems and methods used in many studies. Here, indicate whether each material, system or method listed is relevant to your study. If you are not sure if a list item applies to your research, read the appropriate section before selecting a response.

Materials & experimental systems

n/a	Involved in the study	
☐	☒	Antibodies
☐	☒	Eukaryotic cell lines
☒	☐	Palaeontology and archaeology
☒	☐	Animals and other organisms
☒	☐	Clinical data
☒	☐	Dual use research of concern

Methods

n/a	Involved in the study	
☒	☐	ChIP-seq
☐	☒	Flow cytometry
☒	☐	MRI-based neuroimaging

Antibodies

Antibodies used	Custom mouse monoclonal antibodies against the FUS C-termini were generated at Boster Bio (Pleasanton, CA, USA) with antigens of wild-type P525 (N-DSRGEHRQDRRERPY-C; clone P3-A6C4) or mutant P525L (N-CGKMDSRGEHRQDRRERLY-C; clone P1-E7B6). The custom rabbit anti-FUS (N-terminus) antibody was described previously (PMID: 26250115). The custom rabbit anti-CPSF2 (CPSF-100) antibody was described previously (PMID: 29167381). Commercial antibodies used in this study were: mouse monoclonal anti-FUS 4H11 [Santa Cruz Biotechnology, sc-47711], mouse anti-GAPDH 6C5 [Santa Cruz Biotechnology, sc-32233], rabbit anti-TARDBP [Proteintech Group, 12892-1-AP], mouse anti-TARDBP clone 41-7.1 [Santa-Cruz Biotechnologies, sc-100871], rat anti-TARDBP (phospho-S409/S410) clone 1D3 [Sigma-Aldrich, MABN14], mouse anti-OCT3/4 [Santa Cruz Biotechnology, sc-5279], rabbit anti-NANOG H155 [Santa Cruz Biotechnology, sc-33759], mouse anti-TUBB3 clone Tuj1 [Biolegend, 801202], mouse anti-ISL1/2 clone

	39.4D5 [DSHB, 39.4D5-c], rabbit anti-ISL1 [Abcam, ab20670], mouse anti-Hb9 (MNX1) clone 81.5C10 [DSHB, 81.5C10-c], chicken anti-NFH polyclonal IgY [Millipore, AB5539], mouse monoclonal anti-TOM22 clone 1C9-2 [Sigma-Aldrich T6319-.2ML], rabbit monoclonal anti-MT-CO1 clone EPR19642 [Abcam, ab203917], rabbit polyclonal anti-MT-CO2 [Proteintech 55070-1-AP], rabbit polyclonal anti-MT-CO3 [Sino Biological 202683-T10-100], rabbit polyclonal anti-NDUFA12 [Thermo Scientific PA5-58973], and rabbit polyclonal anti-GAD67 [Sigma ZRB1090]. Secondary antibodies were donkey anti-rabbit IgG IRDye 800CW [Li-Cor, 926-32213], donkey anti-mouse IgG IRDye 800CW [Li-Cor, 926-32212], donkey anti-rabbit IgG IRDye 680LT [Li-Cor, 926-68023], donkey anti-mouse IgG IRDye 680LT [Li-Cor, 926-68022], donkey anti-mouse IgG AlexaFluor 488 [Invitrogen, A-21202], donkey anti-rat IgG AlexaFluor 488 [Invitrogen, A48269 and A21208], donkey anti-rabbit IgG AlexaFluor 546 [Invitrogen, A10040], donkey anti-rabbit IgG AlexaFluor 647 [Invitrogen, A31573], goat anti-rabbit IgG AlexaFluor 488 [Invitrogen, A21206], goat anti-mouse IgG2a AlexaFluor 488 [Invitrogen, A21131], goat anti-mouse IgG2b AlexaFluor 488 [Invitrogen, A21141], goat anti-mouse IgG1 AlexaFluor 568 [Invitrogen, A21124], goat-anti-mouse IgG2a AlexaFluor 568 [Invitrogen, A21134], goat anti-mouse IgG2a AlexaFluor 647 [Invitrogen, A21241], goat anti-mouse IgG2b AlexaFluor 647 [Invitrogen, A21242], donkey anti-chicken IgY AlexaFluor 647 [Invitrogen, A78952], and goat anti-rabbit IgG AlexaFluor 405 [Invitrogen, A31556].
Validation	Commercial primary and secondary antibodies were used according to their specification and validated use as attested by the supplier. Commonly used commercial primary antibodies were chosen against neuronal markers (ISL1, ISL1/2, MNX1, TUBB3), pluripotency markers (NANOG, OCT3/4), as well as against TARDBP targets. The specificities of the FUS antibodies were validated with western blot and immunofluorescence stainings using FUS knockout and mutant lines with protein mislocalization phenotypes as shown in the manuscript. Custom and commercial antibodies used in western blot (FUS, CPSF2; TARDBP, GAPDH) also agreed with the molecular size estimates for the targets. For each immunofluorescence staining, the background fluorescence was controlled by omitting the primary antibodies in one background control sample (included in raw data if recorded).

Eukaryotic cell lines

Policy information about cell lines and Sex and Gender in Research

Cell line source(s)	Human induced pluripotent stem cell line DF6-9-9T.B from WiCell. Derivative lines were generated by genome-engineering as outlined in the manuscript or previously reported for individual mutant cell lines. Human induced pluripotent stem cell KOLF2.1J lines were obtained from The Jackson Laboratory (JAX).
Authentication	PCR amplification of editing on-target and off-target loci followed by dye-terminator sequencing; Immunofluorescence staining for pluripotency markers; Karyotyping with Karyostat service (Thermo Fisher).
Mycoplasma contamination	All cell lines tested negative for mycoplama (PCR testing; MycoAlert mycoplasma detection kit, Lonza).
Commonly misidentified lines (See ICLAC register)	None

Flow Cytometry

Plots

Confirm that:

- [ ] The axis labels state the marker and fluorochrome used (e.g. CD4-FITC).
- [ ] The axis scales are clearly visible. Include numbers along axes only for bottom left plot of group (a 'group' is an analysis of identical markers).
- [ ] All plots are contour plots with outliers or pseudocolor plots.
- [ ] A numerical value for number of cells or percentage (with statistics) is provided.

Methodology

Sample preparation	Adherent neuron cultures were treated with 5 µM Y-27632 (Tocris, 1254) for 1 hour. After washing carefully with HBSS (ThermoFisher, 14025050), the cultures were dissociated in TrypLE Express (ThermoFisher, 12604021), supplemented with 5 µM Y-27632 for 20-30 min under gentle agitation and with pipetting at 5 min intervals to break cell clumps apart. The dissociated neurons were then collected by centrifugation at 200 g for 5 min and resuspended in 500 µL HBSS supplemented with 2% v/v B-27 supplement [ThermoFisher, 0080085SA], 2% v/v KnockOut Serum Replacement [ThermoFisher, 10828028], 2% w/v bovine serum albumin [Sigma-Aldrich, A9418-10G], 25 mM D-glucose [Sigma-Aldrich, G7021-100G], 5 µM Y-27632, 10 ng/mL BDNF [Peprotech, 450-02], 10 ng/mL GDNF [Peprotech, 450-10]. The cell suspension was then filtered through cell strainer caps into FACS tubes (BD Biosciences) and was kept on ice. The single cell suspensions were index sorted within an hour of harvest on a FACSAria III or FACSAria Fusion (BD Biosciences) at 4 °C and 20 psi through a 100 µm ceramic nozzle under sterile conditions. Single cells were collected into 96 multiwell plates (ThermoFisher, 11355960 and 10036339) by dispensing individual cells in 5 nL drops directly into 3 µL ice-cold single cell lysis buffer (scLB, 0.134% Triton X-100 [Sigma], 0.5 U/µL recombinant RNase inhibitor [Takara, 2313B], 3.33 mM each dNTP [ThermoFisher, R1121], 1 mM dithiothreitol [DTT, ThermoFisher], 1.67 µM Smarter-oligo-dTVN [Biomers.net], 6.7e-7 diluted ERCC Spike-In [ThermoFisher, 4456740]). After collection the plates were immediately sealed with adhesive sheets (ThermoFisher, AB0558) and snap frozen on dry ice.
Instrument	FACSAria III and FACSAria Fusion (BD Biosciences), hosted at the Biomedicum Flow Cytometry Core Facility at Karolinska Institutet.
Software	BD FACSDiva software, on site.

Cell population abundance	We did not use fluorescent makers for cell types or neuronal subpopulations during sorting. Instead, cell types were assigned on basis of their single cell transcriptomes and relative abundances were calculated post-hoc.
Gating strategy	Intact cells were separated from crude cellular debris based on size and complexity (FSC x SSC). Gates for single cells were set up in forward (FSC-W x FSC-A) followed by sideward scatter (SSC-W x SSC-A). Dead cells positive for TOPRO-3 iodide were excluded. Gates were set up with a TOPRO-3 iodide-stained live cell control sample and were adjusted with two additional control samples: unstained live cells and TOPRO-3 iodide-stained (ethanol-fixed) dead cells in order to exclude dead cells and neurite debris. All collected populations were affirmed by back-gating.

☐ Tick this box to confirm that a figure exemplifying the gating strategy is provided in the Supplementary Information.
